# Tanshinone IIA inhibits osteoclastogenesis in rheumatoid arthritis via LDHC-regulated ROS generation

**DOI:** 10.1186/s13020-023-00765-1

**Published:** 2023-05-15

**Authors:** Qiuwei Peng, Jian Wang, Man Han, Minghong Zhao, Kesong Li, Tianming Lu, Qiuyan Guo, Quan Jiang

**Affiliations:** 1 Department of Rheumatology, Guang’anmen Hospital, China Academy of Chinese Medical Sciences, Beijing, 100053 China; 2 The First School of Clinical Medicine, Gannan Medical University, Ganzhou, 341000 China; 3 School of Public Health, Guangxi Medical University, Guangxi, 530021 China; 4 Artemisnin Research Center, and Institute of Chinese Materia Medica, China Academy of Chinese Medical Sciences, Beijing, 100700 China

**Keywords:** Tanshinone IIA, Rheumatoid arthritis, Osteoclast, Lactate dehydrogenase, Reactive oxygen species

## Abstract

Rheumatoid arthritis (RA) is characterized by bone destruction in the afflicted joints, and during the process of bone destruction, osteoclasts play a crucial role. Tanshinone IIA (Tan IIA) has shown anti-inflammatory effects in RA. However, the exact molecular mechanisms by which it delays bone destruction remain largely unexplained. Here, we found that Tan IIA decreased the severity of and ameliorated bone loss in an AIA rat model. In vitro, Tan IIA inhibited RANKL-induced osteoclast differentiation. By activity-based protein analysis (ABPP) combined with LC‒MS/MS, we discovered that Tan IIA covalently binds to the lactate dehydrogenase subunit LDHC and inhibits its enzymatic activity. Moreover, we found that Tan IIA inhibits the generation of osteoclast-specific markers by reducing the accumulation of reactive oxygen species (ROS), thus reducing osteoclast differentiation. Finally, our results reveal that Tan IIA suppresses osteoclast differentiation via LDHC-mediated ROS generation in osteoclasts. Tan IIA can thus be regarded as an effective drug for the treatment of bone damage in RA.

**Supplementary Information:**

The online version contains supplementary material available at 10.1186/s13020-023-00765-1.

## Background

Rheumatoid arthritis (RA) is a chronic inflammatory disease characterized by synovitis and bone destruction [1]. The three-year disability rate for RA patients without standardized treatment could be as high as 75% [2]. Once bone destruction has occurred, it is irreversible. Slowing or even stopping the destruction of bone has emerged as a major challenge in the management of RA. However, the mechanisms of bone destruction in RA remain unknown. Studies involving animal models have shown that osteoclast-free arthritis mouse models induced by serum transfer [3] and TNF [4] are entirely unaffected by bone erosion. A growing number of scholars believe that osteoclasts play a crucial role in the progression of bone destruction in RA [5].

Osteoclasts are multinuclear giant cells derived from the monocyte-macrophage lineage that are distributed at the interface between the bone surface and inflammatory synovial tissue [6, 7]. Osteoclast differentiation depends on receptor activator of nuclear factor κB ligand (RANKL), a major osteoclastogenic mediator [8]. Various cytokines and autoantibodies stimulate the activation of osteoclasts and degrade bone matrix by secreting acids and lytic enzymes, thereby promoting bone resorption [9]. Directly targeting osteoclasts in RA can protect bone from inflammation, and osteoclasts can potentially be a promising treatment for RA.

Current medical treatment of RA is mainly based on two main approaches: the administration of nonsteroidal anti-inflammatory drugs and disease-modulating antirheumatic drugs [10]. However, most of the current drugs are mainly used to control pain and joint synovitis inflammation and interfere little with osteoclasts. Moreover, the side effects, high cost, and toxicity of long-term use of drugs are unavoidable [11]. Therefore, research on the treatment of RA with Chinese medicines and Chinese medicine monomers is increasing.

Tanshinone IIA (Tan IIA) is a natural product derived from the plant *Salvia miltiorrhiza* [12] that has been mainly used for treating insomnia, cardiovascular disease and menorrhalgia [13, 14]. Due to its anti-inflammatory and proapoptotic activities, studies on its anti-RA mechanism have attracted increasing attention [15–17]. Tan IIA was reported to regulate lncRNA GAS5 to promote apoptosis in fibroblast-like synoviocytes (FLSs) [15]. Tan IIA also promoted RA-FLS apoptosis by altering the G2/M phase of the cell cycle [17]. A clinical study investigated the effect of Tan IIA in patients with RA, and the results demonstrated that Tan IIA reduced the levels of various inflammatory factors to induce an anti-inflammatory response [18]. To date, some progress has been made in explaining the anti-RA activity mechanisms of Tan IIA. However, the mechanisms underlying the roles of Tan IIA in RA remain largely elusive.

The aerobic glycolytic pathway is necessary to maintain the energy required for osteoclast differentiation [19]. RA monocytes exhibit elevated levels of glycolytic enzymes whose blockade eliminated proinflammatory mediators. The phenotype described above precedes the onset of clinical disease [20]. After differentiation into mature ex vivo polarized macrophages, this phenotype was maintained. Such activation of proinflammatory pathways corresponds to alterations in cellular bioenergetics [21]. CD4 T cells promote the invasiveness of and glycolysis in FLSs, whereas glycolysis inhibitors prevent this activation [22]. The manipulation of glycolysis has key clinical implications for treating RA. Glycolysis strongly depends on the metabolic enzyme LDH, which facilitates the glycolytic process by converting pyruvate to lactate [23]. Previous research has demonstrated that upregulating LDH activity promotes glycolysis, consequently potentiating mature osteoclast formation via nuclear factor of activated T-cell (NFAT) c1 signalling during osteoclastogenesis [24].

The purpose of this study was to describe the effect of Tan IIA on bone destruction in RA by investigating how Tan IIA ameliorates bone destruction and exploring the therapeutic potential of targeting osteoclast metabolism. Tan IIA candidate target proteins were identified using activity-based protein profiling (ABPP).

## Methods

### Reagents and chemicals

Tan IIA was purchased from Beijing Bethealth People Biomedical Technology (Beijing, China; purity ≥ 98%). Complete Freund's adjuvant (CFA) was purchased from Becton, Dickinson and Company (USA). Foetal bovine serum was obtained from Corning (USA), and penicillin/streptomycin was obtained from Thermo Fisher (USA). RANKL was obtained from Novoprotein (Suzhou). Cell fixative was obtained from Biorigin (Beijing). The Tartrate-Resistant Acid Phosphatase (TRAP) Stain Kit was obtained from Solarbio (Beijing). Actin-Tracker Red-Rhodamine was obtained from Beyotime Biotechnology (Beijing). A lactate dehydrogenase assay kit was obtained from Solarbio (Beijing). The NAD/NADH assay kit was obtained from BioXcellence (Beijing). Rabbit anti-mouse IgG was obtained from Abcam (Beijing). The desthiobiotin iodoacetamide (DBIA) probe was obtained from ChomiX Biotech (Nanjing). The following click chemistry reaction and LC‒MS/MS reagents were used in this study: TBTA (1770049), TCEP (C4706), rhodamine-N3 (83689) and CuSO4 (C1297) were purchased from Sigma (USA); TMT 10plex™ reagent (A34808), high-capacity neutravidin agarose resin (A53031) and sequencing grade modified trypsin (90057) were purchased from Thermo Fisher (USA).

### Adjuvant-induced arthritis (AIA) rats and Tan IIA treatment

The Ethics Committee of the Chinese Academy of Chinese Medical Sciences approved all animal experiments (ethics number: 2022B115). Healthy male Lewis rats (6–8 weeks old, 170–200 g) were purchased from Beijing Charles River Laboratories (SCXK (Beijing) 2021–0011). They were maintained under standard conditions for 7 days before any manipulations. The 36 rats were randomly allocated to six groups (6 rats per group): AIA model group, normal control group, Tan IIA high, middle and low dosage groups (30 mg/kg/d, 15 mg/kg/d, 7.5 mg/kg/d) and methotrexate (MTX) group (0.2 mg/kg, twice weekly). The method of AIA induction was performed based on previously reported literature [25]. With the exception of the rats in the normal control group, all rats were injected with 0.1 mL CFA to induce arthritis. The rats in the normal control group were injected with an equivalent amount of saline instead. Treatment was administered continuously for 30 days after AIA model construction. The rats in the Tan IIA treatment groups and MTX group received a single daily gavage of the appropriate drug twice weekly. The rats in the normal control and AIA model groups received an equal volume of distilled water instead.

### Arthritis evaluation

Paw Swelling: the ipsilateral hindfoot thickness of the rats in each group was measured using Vernier callipers as previously reported, and these measurements were repeated three times [26]. The frequency of disease symptoms in each group of rats independent of severity indicated morbidity [27]. Clinical arthritis scores were assessed by two individuals who were blinded to the experimental groups [28]. Based on a previous report [29], the rats’ pain domain was measured with a Von Frey hair mechanical pain tester.

### Histopathological evaluation of the joints

Rat right knee joints were fixed for 48 h in 4% paraformaldehyde before being decalcified for 6 weeks in 10% ethylenediaminetetraacetic acid (EDTA). The rats’ right knee joints were paraffin-embedded, sliced, and stained with haematoxylin–eosin (HE) after being completely decalcified. Histopathological scoring was performed as previously reported [30]. Histomorphological changes in the articular cartilage were observed by light microscopy. Three independent blinded pathologists assessed and quantified the histopathological evaluations.

### Micro-CT

Microfocal computed tomography (Skyscan 1276, Bruker, Belgium; resolution, 10.2 µm) was used to scan the rats’ left paws, ankles, and knees. An image analysis device (CTan V 1.13 software, Bruker micro-CT) was used to recreate and analyse the scanned pictures. This system can also provide three-dimensional spatial structural parameters of the bone trabeculae in the monitored area, including the bone volume (BV)/total volume (TV) ratio, trabecular number (Tb. N), bone surface ratio (BS/BV), and trabecular thickness (Tb. Th).

### Enzyme-linked immunosorbent assay

At the end of the experiment, blood was collected from the rats under general anaesthesia. Rat sera were obtained by centrifugation at 4 °C for 15 min (3000×). The sera were examined for relevant cytokine bone resorption indicators according to the MMP-9, TRAP, CTSK and interleukin 17 (IL-17) ELISA kit instructions.

ELISA was used to measure the effect of the LDHC inhibitor galloflavin (GF) on the osteoclast-specific indices MMP-9, TRAP, CTSK and IL-17. Rats were divided into the control group, Tan IIA (20 μM) group, GF (20 μM) group, and GF (20 μM) + Tan IIA (20 μM) group.

### Cell culture

RAW264.7 cells were obtained from the Chinese Academy of Medical Sciences (Beijing, China). All cells were grown in a 5% CO2 incubator at 37 °C and cultured in glucose-containing DMEM supplemented with 10% FBS and 100 IU penicillin/streptomycin. Cells were passaged at 1:3 when they were in the logarithmic growth phase and at a density of approximately 90%.

### Osteoclast differentiation

RAW264.7 cells (2.5 × 10^5^) were inoculated in 24-well plates and cultured in full DMEM with 50 ng/mL RANKL. The incubator conditions were set to 37 °C and 5% CO_2_. Cell growth was observed until mature osteoclasts had formed.

### TRAP staining assay and actin ring immunofluorescence

Osteoclasts were cultured in 24-well plates for 20 min before being washed with PBS and fixed with tissue cell fixative. The osteoclasts were then stained using the TRAP Stain Kit as directed by the manufacturer. Osteoclasts, defined as TRAP-positive multinucleated cells (nuclei ≥ 3), were photographed for observation of cell morphology with an ECHO microscope (200×), and the osteoclast fusion index was calculated. The induced osteoclasts were permeabilized with 0.1% Triton X-100 for 10 min and washed twice with PBS. The cells were then incubated at room temperature for 1 h with Actin-Tracker Red-Rhodamine (1:100 dilution) and Hoechst (1:500 dilution) staining solutions. A confocal fluorescence microscope was used to observe the cells (Leica TCS SP8 SR).

### LDHC enzyme assay

Enzyme activity was measured using a lactate dehydrogenase assay kit. Tan IIA was added to 0.5 μg/μL LDHC (10 μL) in PBS, and the absorbance at 340 nm was measured to determine the enzyme activity. Scans were performed using a microplate reader every 2 min until 36 min (see Additional file 1).

### NAD^+^/NADH ratio

Intracellular NAD^+^/NADH was measured following the instructions of the NAD/NADH assay kit. Osteoclasts (1 × 10^6^ cells/well) were placed in a 6-well culture plate treated with DMEM with or without Tan IIA (10, 20, 40 μM) for 24 h before being washed with PBS. The NAD^+^/NADH assay was performed according to the kit instructions. The absorbance values at 570 nm were measured using a microplate reader (EnVision 2105, PerkinElmer, USA), and the total amounts of NAD^+^ and NADH were estimated. According to the formula below, the ratio of NAD^+^/NADH in the sample was calculated. [NAD^+^/NADH] = [NAD^+^]/([NADH total]-[NADH]).

### Cellular imaging

Fluorescence imaging assessments demonstrated the colocalization of Tan IIA with the potential cell target LDHC. In the presence or absence of Tan IIA (0 and 20 μM), osteoclasts were treated with 0.5 mL of 10% DMEM. IAA (40 μM) was added to the medium for 1 h after 4 h of DMEM treatment. Next, 1 mL of PBS was used to wash the cells. After that, the cells were fixed for 20 min with cell fixative. Triton X-100 (0.1%) was applied to permeabilize the cells for 10 min. Bovine serum albumin (BSA, 5%) was added for 2 h at room temperature. An LDHC antibody (1:1000) was applied for overnight incubation at 4 °C. Rabbit anti-mouse IgG (1:1000) (Alexa Fluor 488) was then added for incubation at room temperature for 1 h after washing with TBST. Finally, the cells were treated for 30 min with Hoechst (1:500 dilution) before being imaged with a laser scanning confocal fluorescence microscope (Leica TCS SP8 SR).

We used the probe DCF-DA to detect ROS accumulation in osteoclasts. Briefly, cells (1 × 10^5^/well) were inoculated in laser confocal dishes and treated with Tan IIA (0, 10, 20, 40 μM) for 12 h. Cells were then incubated with 10 mM DCF-DA and Hoechst (1:500 dilution) for 30 min and observed under a confocal fluorescence microscope (Leica TCS SP8 SR).

### Competitive in-gel fluorescent labelling of Tan IIA in osteoclasts

Osteoclasts (2 × 10^6^ cells/well) were grown to 90% confluence on 6-cm plates before being exposed to different concentrations of Tan IIA (0, 50, 100, and 200 400 μM). Cells were then washed twice with PBS. With the use of a cell scraper, cells were collected. Cell lysate solution (100 μL RIPA with 1% protease inhibitor) was then added, and the cells were sonicated on ice until protein lysis was achieved. Following centrifugation at 15,000 ×*g* for 15 min, the protein lysate supernatant was aspirated. Using a BCA kit, the protein concentration was adjusted to 2 mg/mL. Two hundred micrograms of cell lysate was collected, and 50 μM cysteine-specific probe (IAA-yne) was added for labelling. Next, each sample was added to 13 μL of click buffer containing 6 μL of TBTA (1.7 mM, DMSO), 2 μL of CuSO4 (50 mM, ddH2O), 0.6 μL of biotin-N3 (10 mM), and 2 μL of TCEP (50 mM, ddH_2_O) and shaken at 1000 rpm at 29 °C for 1 h. One millilitres of precooled acetone (− 20 °C) was added to the mixture, and the marker proteins were precipitated for 30 min at − 80 °C. After 15 min of centrifuging the supernatant at 15,000 rpm, the acetone was allowed to evaporate completely. The protein samples were prepared by adding 100 μL of 1 × loading buffer and heating in a metal bath at 96 °C for 10 min. Protein samples were separated on 10% SDS‒PAGE gels. Total proteins were visualized using Coomassie brilliant blue. Fluorescence gel images were acquired with an Azure Sapphire RGB NIR scanner (USA).

### Streamlined cysteine activity-based protein profiling

To find the cellular proteins that interact with Tan IIA based on activity, we performed competitive mass spectrometry experiments. In the following steps, we chose the probe DBIA. Three groups were assembled: 500 μM DBIA, 100 μM Tan IIA + 500 μM DBIA and 200 μM Tan IIA + 500 μM DBIA. Tan IIA was used for the competition experiments. The protocols for osteoclast culture, Tan IIA treatment, protein collection, and DBIA labelling were identical to those previously reported for the Tan IIA treatment of osteoclasts in an in-gel fluorescent labelling experiment. After adding 500 μM DBIA to 100 μL of Tan IIA-treated cell lysate, the cells were shaken for 1 h at 1000 rpm and 29 °C. DTT was added at a final concentration of 5 mM after probe labelling, and the cells were shaken for 30 min (1000 rpm, 29 °C) in the dark. To reduce cysteine residues, 20 mM IAA was added with shaking (1000 rpm, 29 °C) at the end of the reaction and in the dark for 30 min. Subsequently, to remove as much DBIA as possible, the proteins were precipitated using a chloroform/methanol/water combination (1:4:3) and centrifuged at 15,000 rpm for 15 min. After centrifugation, the supernatant was gently aspirated using a pipette, and the proteins were washed and precipitated twice with 500 μL of methanol. For peptide digestion, 200 μL of 200 mM EPPS (pH 8.52) was used to redissolve the precipitate, while 2 μL of trypsin and LysC (1 μg/μL, Promega) were added to digest the proteins. The reaction was carried out on a shaker for 20 h protected from light (1000 rpm, 29 °C). For peptide labelling, peptides digested with DBIA-conjugated cysteine were tagged using TMT10plex™ mass labelling reagent (Thermo Scientific) following the manufacturer s instructions. After 15 min of incubation with 0.3% hydroxylamine, the TMT reaction was stopped. After mixing all of the TMT-labelled samples with a SpeedVac, they were dried. The samples were dissolved in 1 mL of PBS for peptide enrichment, 100 μL of streptavidin magnetic beads were added, and the samples were swirled at room temperature for 4 h. The beads were washed three times with 1 of mL PBS, 1 mL of 0.1% SDS in a SpeedVac, and 1 mL of dd H_2_O was added to eliminate nonspecific binding. Finally, after desalting with 0.1% formic acid, the DBIA-labelled peptides were eluted from a C18 column with 0.1% FA in 50% acetonitrile, and the samples were analysed using an Orbitrap Fusion Lumos (Thermo Scientific).

### Whole-proteome experiment

First, tissues from the ankle joint cavities of the rats in the normal control, model, and Tan IIA high-dose (30 mg/kg) groups were lysed as previously reported. Protein concentrations were measured and diluted to a concentration of 3 mg/mL. Second, at room temperature, 5 mM DTT was added to samples for 30 min of treatment in the dark. Then, 20 mM IAA was added for 30 min at room temperature in the dark. The proteins were precipitated using a chloroform/methanol/water system (1:4:3). After that, the precipitated protein was dissolved in EPPS buffer. To digest the protein, 1 μg/μL trypsin was added for 20 h at 37 °C. Samples were desalted using a commercial C18 column (Waters) and dissolved in 0.1% FA for LC‒MS/MS identification.

### Statistical analysis

GraphPad Prism 9.0 software was used for statistical analysis. *T* tests, one-way ANOVA, Welch’s test, Brown-Forsythe test, and nonparametric tests were employed to analyse the data. P values < 0.05 were considered to indicate statistical significance.

## Results

### Tan IIA significantly ameliorated the severity of RA

**Fig. 1:**
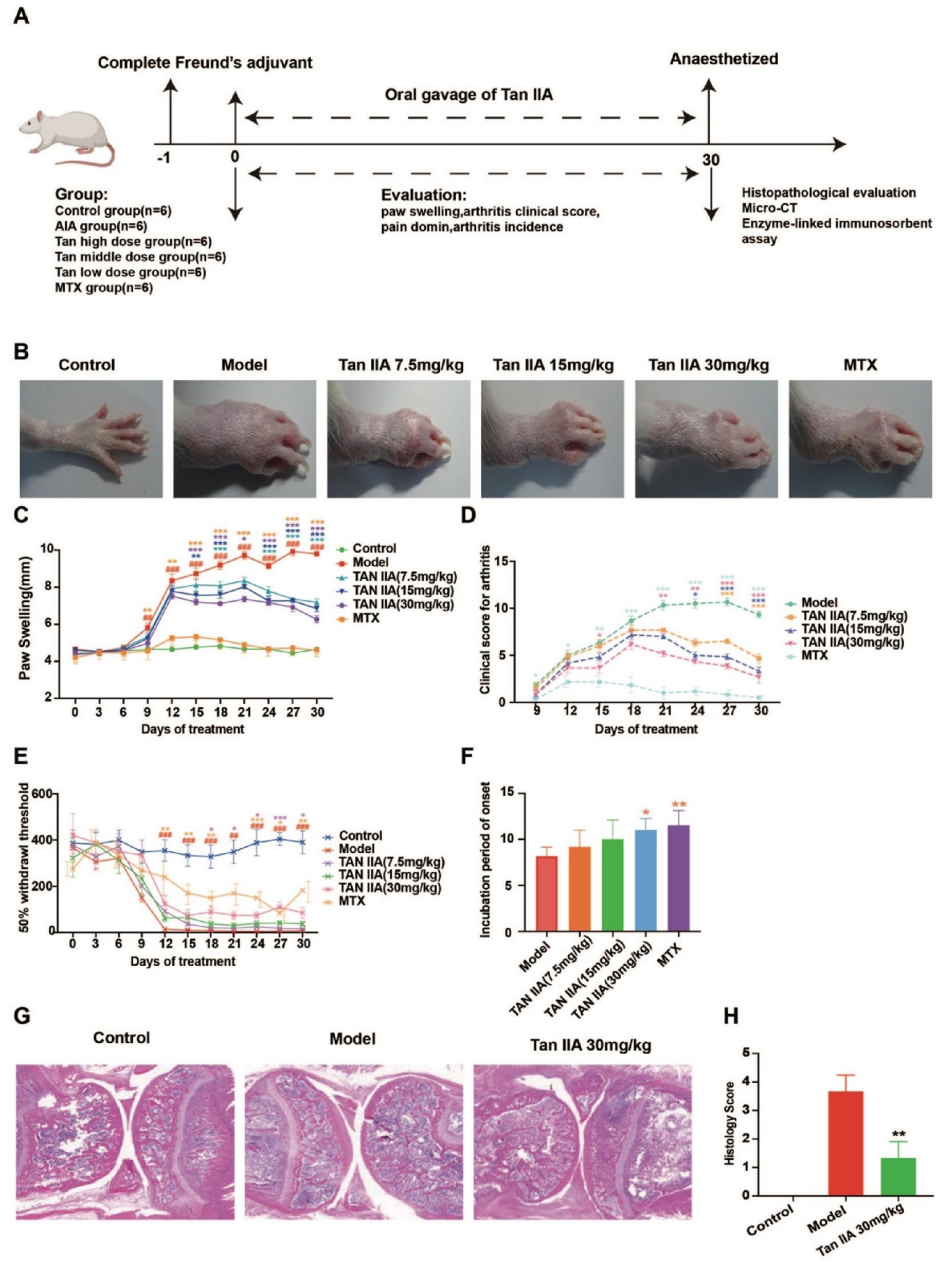
The effect of Tan IIA on the severity of RA in AIA rats. **A** The design of the animal experiment. **B** Representative photographs of arthritis symptoms in rat hind paws. **C** The assessment of paw swelling (data are mean ± SD, n = 6, ^##^*P* < 0.01, ^###^*P* < 0.001 vs. Control; ^*^P < 0.05, ^**^P < 0.01, ^***^P < 0.001 vs. Model). **D** Evaluation of clinical arthritis scores (data are mean ± SD, n = 6, ^*^P < 0.05, ^**^P < 0.01, ^***^P < 0.001 vs. Model). **E** Measurement of the pain domain (data are mean ± SD, n = 6, ^###^*P* < 0.001 vs. Control; ^*^P < 0.05, ^**^P < 0.01, ^***^P < 0.001 vs. Model). **F** The percentage of animals exhibiting any sign of disease (data are mean ± SD, n = 6, ^*^P < 0.05, ^**^P < 0.01 vs. Model). **G** Typical images of H&E staining (Bar = 50 μm) of the knee joints. **H** Histological scores (data are mean ± SD, n = 6, ^**^P < 0.01 vs. Model)

We first assessed the influence of Tan IIA on the severity of RA in AIA rats. The design of the animal experiment is depicted in Fig. 1A. Tan IIA reduced paw swelling, clinical arthritis scores and the incidence of arthritis in AIA rats in a dose-dependent manner (Fig. 1C, D and F). In the model group, we observed significant arthritic symptoms, such as erythema and swelling, in the acquired photographs, while Tan IIA significantly reduced the arthritic symptoms of AIA (Fig. 1B). We also focused on the assessment of pain, which is a common clinical symptom of RA. The results showed that the rats in the Tan IIA high dose group (30 mg/kg) had an increased pain threshold compared with the model group (*P* < 0.05) (Fig. 1E). We observed the pathological changes in joint tissue by microscopy and calculated the pathological scores. The rats in the model group showed obvious joint soft tissue and synovial hyperplasia, inflammatory cell infiltration, joint opacity formation and joint space narrowing (Fig. 1G). Compared with the model group, the rats in the Tan IIA high dose group (30 mg/kg) displayed effectively reduced histological severity scores (*P* < 0.01) (Fig. 1H).

### Tan IIA ameliorated bone destruction in AIA rats

**Fig. 2:**
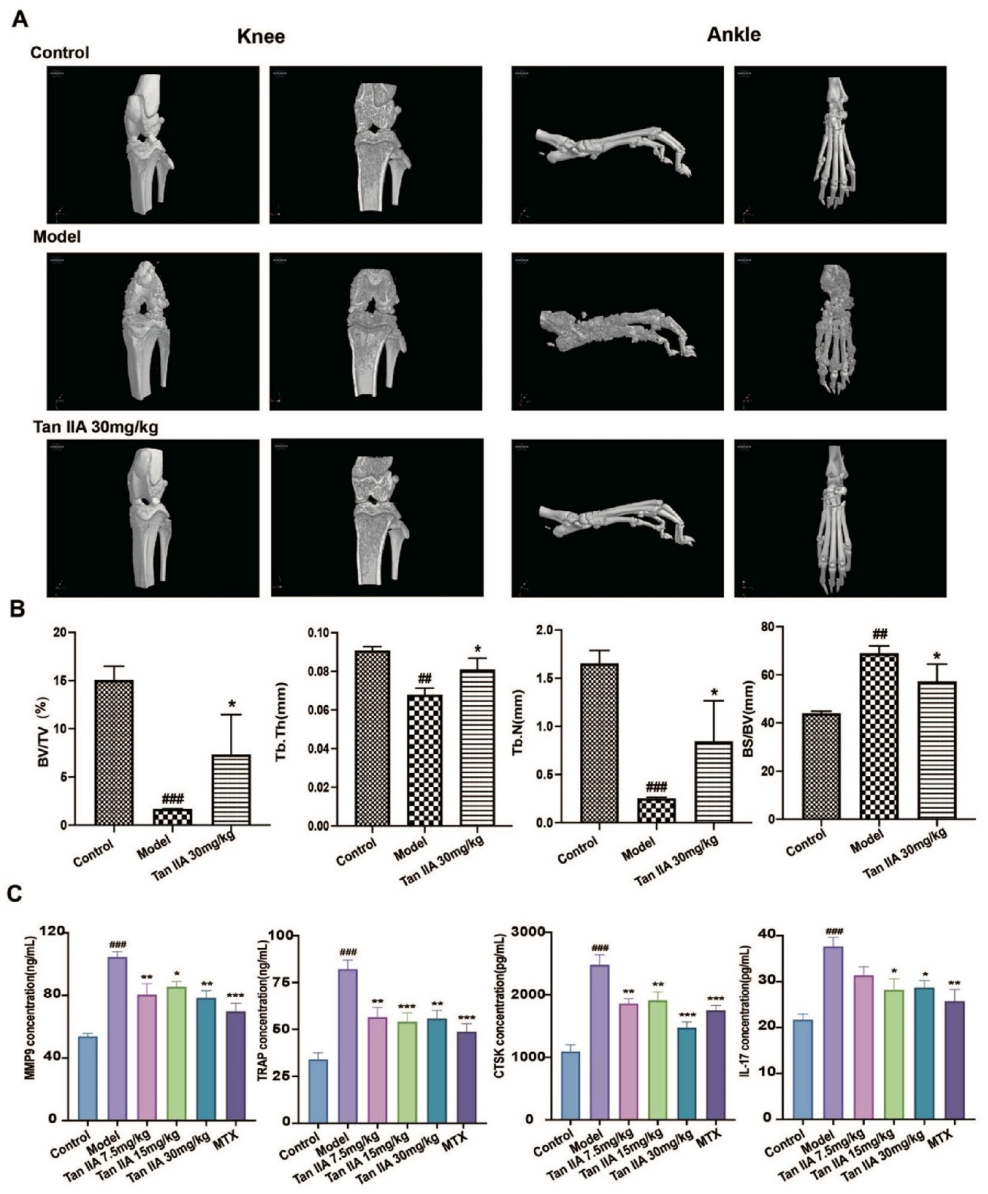
The effect of Tan IIA on bone destruction in AIA rats. **A** Micro-CT images of the knees and ankles. **B** Parameters of the knee joints (data are mean ± SD, n = 6, ^##^P < 0.01, ^###^P < 0.001 vs. Control; ^*^P < 0.05 vs. Model). **C** The effect of Tan IIA on the levels of MMP-9, IL-17, TRAP and CTSK in AIA rats (data are mean ± SD, n = 6, ^###^P < 0.001 vs. Control; ^*^P < 0.05, ^**^P < 0.01, ^***^P < 0.001 vs. Model)

Micro-CT was used to investigate the effect of Tan IIA on bone and joint destruction (Fig. 2A). Compared to those in the normal control group, severe joint injury and bone loss with joint space narrowing were observed in the AIA model rats. In contrast, Tan IIA (30 mg/kg) treatment significantly attenuated joint destruction. In addition, we assessed the quantitative histomorphometric structural bone parameters to measure the degree of bone destruction. The BV/TV, Tb. Th and Tb. N values decreased and BS/BV increased in the model group compared to the normal control group. Tan IIA treatment (30 mg/kg) significantly increased BV/TV, Tb. Th and Tb. N and decreased BS/BV compared to the AIA group (*P* < 0.05) (Fig. 2B). To further demonstrate the effect of Tan IIA on systemic bone metabolism, the levels of the bone resorption-related indicators MMP-9, TRAP, CTSK, and IL-17 were assayed in rat sera (Fig. 2C). Consistent with the radiological results, the contents of MMP-9, TRAP, CTSK, and IL-17 were high in the model rats compared with those in the control group (*P* < 0.001), while these levels trended in the opposite direction in AIA rats treated with Tan IIA (*P* < 0.05) (Fig. 2C).

### Tan IIA suppressed osteoclast markers while inhibiting RANKL-induced osteoclast differentiation and bone resorption in vitro

**Fig. 3:**
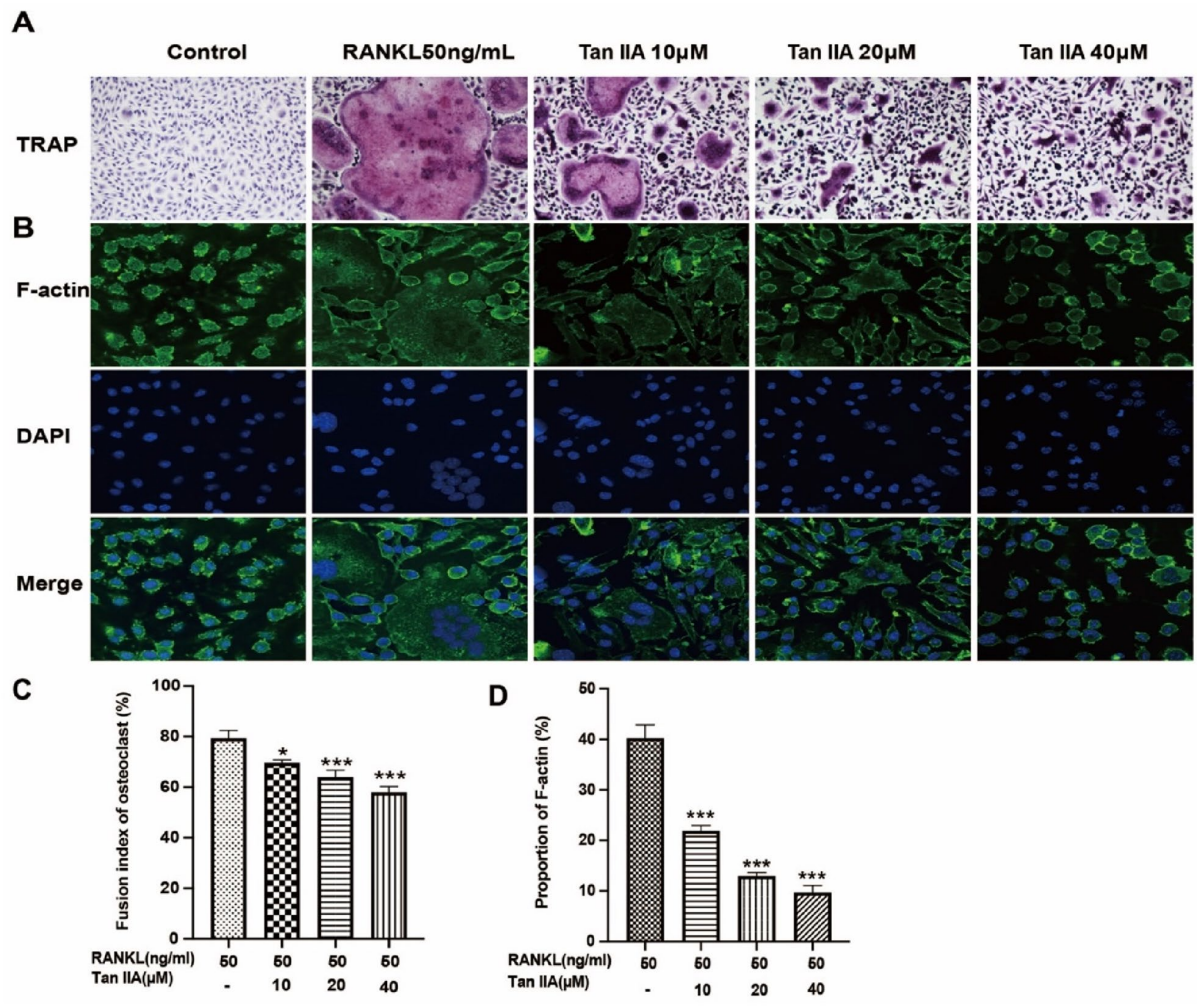
The effects of Tan IIA on RANKL-induced osteoclast differentiation and bone resorption in vitro. **A** Anti-tartrate phosphatase (TRAP)-stained sections of RANKL-induced osteoclasts. **B** Detection of RANKL-induced actin ring formation in osteoclasts. Immunofluorescence staining was used to identify the actin rings (red). Nuclei were stained with DAPI (blue). **C** Fusion indices of osteoclasts (multinucleated TRAP-positive cells). **D** The proportion of actin ring formation (data are mean ± SD, n = 3 for **C** and **D**, ^*^P < 0.05, ^***^P < 0.001 vs. RANKL group)

The influence of Tan IIA on RANKL-induced osteoclast formation was next assessed in vitro. After 5 days of incubation with the RAW 264.7 cells in the control group, TRAP staining was performed, and relatively homogeneous mononuclear macrophages were observed (Fig. 3A). In contrast, in RAW 264.7 cells exposed to 50 ng/mL RANKL for 5 days, a large number of TRAP-positive multinucleated cells (no less than 3 nuclei) were observed, which also had significantly larger volumes, burgundy-coloured cytoplasm, uneven envelope borders and extended surrounding pseudopods. The cells in the Tan IIA-treated groups (10, 20, and 40 μM) showed significantly reduced osteoclast activity in a dose-dependent manner compared to those in the RANKL group (*P* < 0.05) (Fig. 3C). Actin rings are a component of the osteoclast skeleton and play an essential role in the activity of mature osteoclasts. As illustrated in Fig. 3B, there were no osteoclasts or obvious actin ring formation in the control group. In contrast, apparent osteoclasts and actin ring formation were observed in the RANKL-induced group, and many nuclei were observed in the osteoclasts. The rate of actin ring formation was significantly reduced in the Tan IIA-treated groups (10, 20, and 40 μM) compared to the RANKL group (*P* < 0.001) (Fig. 3D).

### Tan IIA directly targets LDHC

**Fig. 4:**
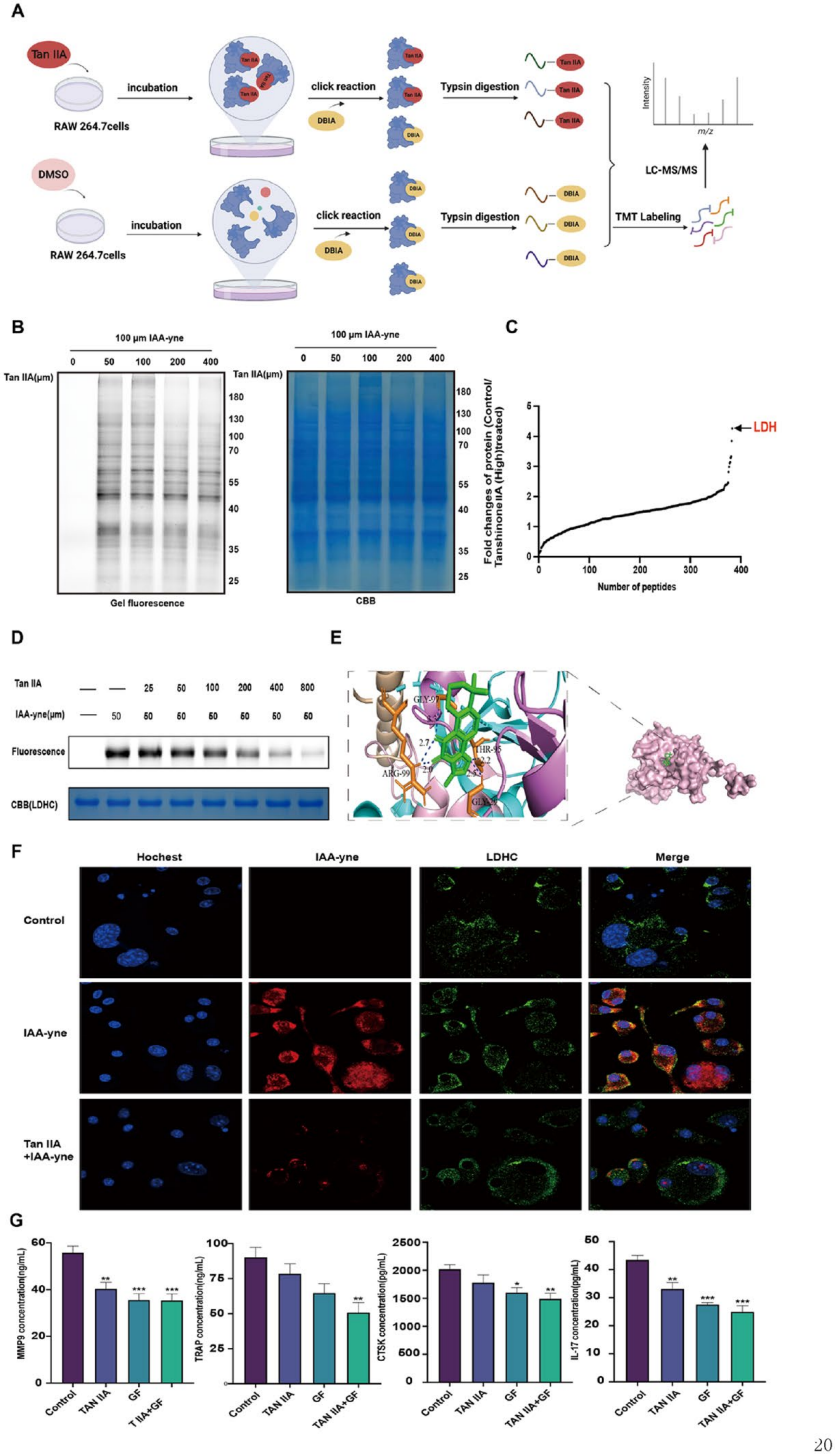
Identification and validation of the target of Tan IIA through ABPP in combination with LC‒MS/MS. **A** The workflow of the ABPP approach for exploring potential Tan IIA targets. **B** Competition of protein labelling with IAA-alkynyl after RAW 264.7 cells were treated with Tan IIA. **C** Identification of potential targets of Tan IIA in RAW 264.7 cells by chemical proteomics. **D** Tan IIA competes with IAA-alkynyl for binding to the purified recombinant LDHC protein, as determined by an in-gel fluorescence assay. **E** Molecular docking simulation of Tan IIA binding to the LDHC protein. **F** Immunofluorescence staining of LDHC (green) and IAA-alkynyl clicked with the red fluorescence dye TAMRA (Bar = 10 μm). **G** An LDHC scavenger inhibited the expression of osteoclast-specific markers (data are mean ± SD, ^*^P < 0.05, ^**^P < 0.01, ^***^P < 0.001 vs. Control)

To identify the potential target proteins of Tan IIA, we adopted ABPP technology. The flow chart of the ABPP experiment is shown in Fig. 4A. Tan IIA dose-dependently competed with the proteins labelled by IAA-alkynyl in osteoclasts (Fig. 4B). The in situ-labelled protein samples treated with Tan IIA were then processed and evaluated by mass spectrometry. As shown in Fig. 4C, LDHC was further investigated because it had the highest fold competition value. We utilized the alkyne tag to perform immunofluorescence staining to examine Tan IIA and LDHC subcellular localization. Tan IIA competed with IAA-alkynyl labelling in osteoclasts (Fig. 4F). Similar results were observed when the purified recombinant LDHC protein was used in the assay (Fig. 4D). In addition, we analysed the binding site of Tan IIA to LDHC by molecular docking analysis, and the results showed that Tan IIA binds to the residues GLY-97, ARG-99, THR-95 and GLY-29 of LDHC (binding energy: − 6.46 kcal/mol) (Fig. 4E), a result which needs further experimental confirmation. Compared with the control group, the LDHC scavenger GF combined with Tan IIA effectively decreased the levels of the osteoclast-specific markers MMP-9, TRAP, RANKL and IL-17, as detected by ELISA (*P* < 0.01) (Fig. 4G).

### Tan IIA inhibits the enzymatic activity of the LDHC protein

**Fig. 5:**
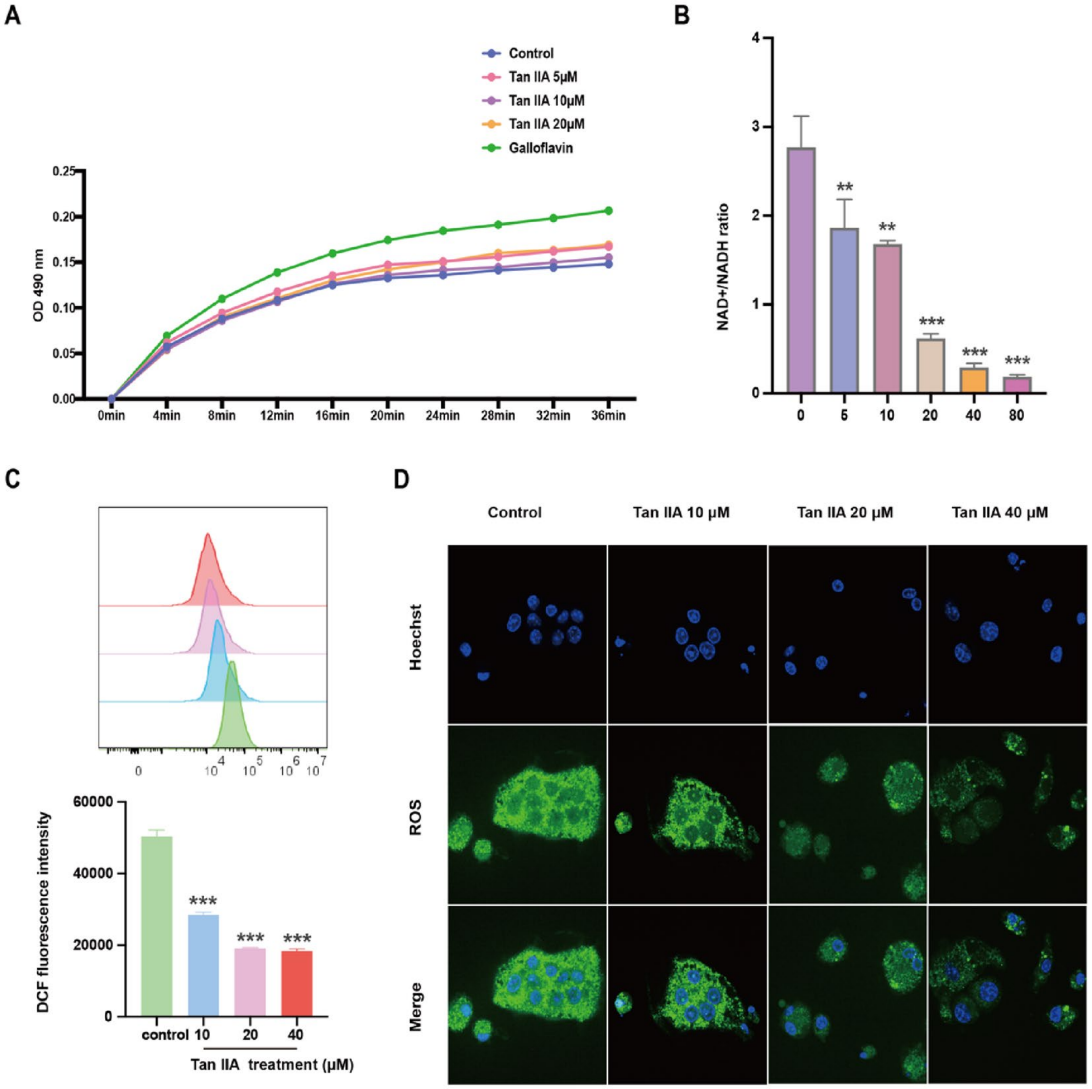
Tan IIA affects the enzymatic activity of the LDHC protein in vitro. **A** Tan IIA inhibits the enzymatic activity of the LDHC protein. **B** Tan IIA reduced NAD production in RAW 264.7 cells (data are mean ± SD, ^**^P < 0.01, ^***^P < 0.001 vs. Control). Tan IIA induced ROS levels in RAW264.7 cells, as determined by flow cytometry (data are the mean ± SD, ^***^P < 0.001 vs. control) (**C**) and immunofluorescence (bar = 50 μm) (**D**)

A prerequisite for sustained glycolysis is that NADH must be continuously oxidized to NAD^+^. LDHC plays the key role in the inhibition of oxidative phosphorylation. LDHC catalyses the production of lactate from pyruvate while simultaneously oxidizing NADH to NAD^+^. We found that Tan IIA could inhibit the enzymatic activity of LDHC (Fig. 5A). Correspondingly, the generation of NAD^+^ was significantly decreased after Tan IIA treatment (*P* < 0.01) (Fig. 5B). Since the enzymatic activity of LDHC is related to oxidative stress, we used flow cytometry and immunofluorescence analyses to evaluate the cellular ROS levels in osteoclasts. The results demonstrated that after Tan IIA treatment, the ROS level significantly decreased compared to that in the control group (*P* < 0.001) (Fig. 5C and D).

### Tan IIA affects metabolic regulation as determined by proteomic analysis

**Fig. 6:**
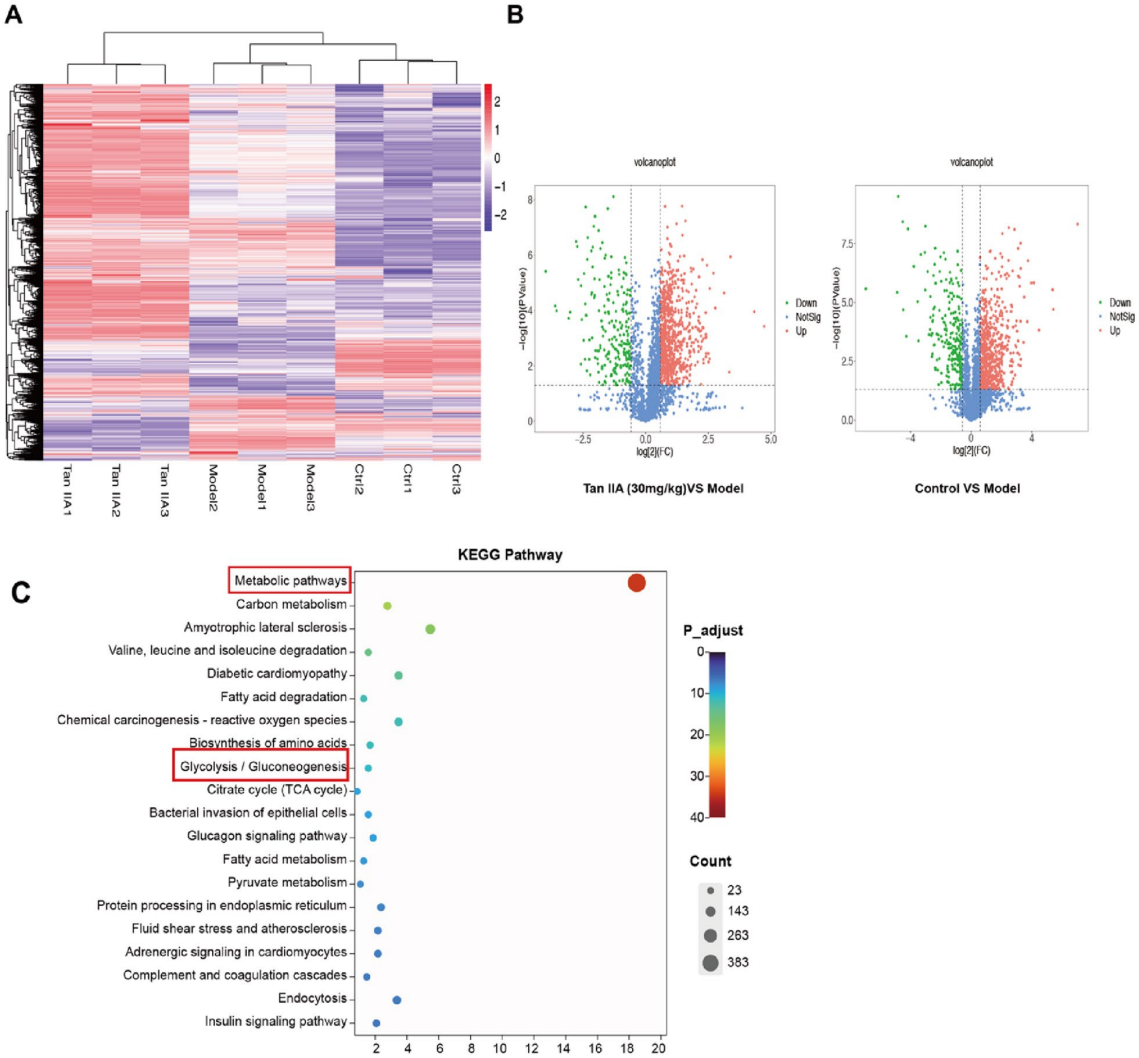
Proteomic analysis of Tan IIA-induced RAW 264.7 cells. **A** Heatmap of the DEGs between the Tan IIA high dose (30 mg/kg/d), model and control samples. Red rectangles represent high expression, and purple rectangles represent low expression. **B** Volcano plots of the DEGs between the Tan IIA high dose (30 mg/kg), model and control samples. Red points represent upregulated genes, and green points represent downregulated genes. **C** KEGG pathway analysis of the DEGs between the Tan IIA high dose (30 mg/kg), model and control samples. The top 20 enriched KEGG pathways

Proteomic analysis was performed to explore the influence of Tan IIA on cellular metabolic regulation. The volcano plot results showed that there were 676 upregulated proteins and 228 downregulated proteins in the control vs. model group (Fig. 6B). In the Tan IIA high-dose group (30 mg/kg) vs. the model group, 785 proteins were upregulated, and 217 were downregulated (Fig. 6B). The intersections of the two groups of differential proteins are displayed as a heatmap (Fig. 6A) and were selected for subsequent KEGG enrichment analysis. The top 20 enriched KEGG pathways are shown in Fig. 6C, and the results indicated that these proteins were associated with the metabolic pathway and glycolysis pathway.

## Discussion

Bone erosion is the main pathological feature of rheumatoid arthritis. The main function of osteoclasts is to balance bone resorption and bone formation, and disruption of this balance leads to bone damage and bone metabolism disorders in RA [31]. Tan IIA suppressed LPS-mediated bone loss [32] and bone loss in experimental mice [33]. These results are in accordance with our findings. We used an AIA model to observe the effects of Tan IIA on severity of RA and bone loss. The results demonstrated that Tan IIA decreased the clinical arthritis scores, arthritis incidence and pathological scores. Tan IIA increased the pain threshold of AIA rats to mechanical and cold stimulation. Tan IIA suppressed bone loss and inhibited bone destruction according to the micro-CT results. In in vitro studies, Tan IIA reduced the formation of osteoclasts, which was in line with the in vivo results. Overall, our findings show that Tan IIA can potentially and effectively ameliorate bone destruction in RA.

Tan IIA has been shown to downregulate c-fos and NFATc1 expression in osteoclast-related bone disease, inhibiting bone loss [34]. However, the direct target of the effects of Tan IIA on osteoclast differentiation in RA is unknown. To discover the potential mechanism and cellular target protein of Tan IIA, we used the probe IAA-yne. Subsequently, the target proteins to which Tan IIA covalently bound were identified by ABPP and LC‒MS/MS analyses. The ABPP results confirmed that Tan IIA could bind directly to LDHC in RANKL-induced osteoclasts. Enzyme activity assays showed that Tan IIA inhibited LDHC activity. Immunofluorescence assays showed that Tan IIA directly targets LDHC.

Lactate dehydrogenase (LDH), in a family of 2-hydroxyacid oxidoreductases, is a tetrameric enzyme consisting of four subunits [35]. The two most common proteins are LDHA and LDHB, with LDHC being the third subunit that has been shown to be distributed in germ cells [36]. Recent studies have focused on the functions of LDH in cancer and metabolic syndromes, including diabetes [37]. Elevated glycolysis is a primary characteristic of osteoclast differentiation, as glucose is the primary energy source in osteoclasts [19]. Glycolysis strongly depends on metabolic enzymes. LDH facilitates the glycolytic process by converting pyruvate to lactate [23]. This information matches well with the results of the proteomic analysis, in which Tan IIA-regulated proteins were associated with metabolic pathways and glycolysis pathways. Previous research has demonstrated that LDH is highly expressed in osteoclast progenitors, which is related to osteoclastogenesis [24]. In our study, Tan IIA suppressed osteoclast formation by inhibiting LDHC activity, and the LDHC scavenger effectively reduced the levels of MMP-9, TRAP, RANKL and IL-17.

LDH is an important enzyme involved in catalysing the redox reaction between lactate and pyruvate in the process of glycolysis [35, 38]. Some reports have shown that high LDH activity is a hallmark of cancer, in which the regulation of ROS plays a key role [23, 39]. ROS, important products of oxidative damage, may be important in promoting osteoclast differentiation [40]. In chondrocytes in an inflammatory state, LDHA can promote ROS formation, while inhibition of LDHA activity can exert an anti-inflammatory effects [41]. Our results show that Tan IIA decreased the ROS levels in osteoclasts by flow cytometry and immunofluorescence. There are few studies on the association between LDH and ROS in osteoclasts. This is the first work to provide a mechanistic explanation of the relationship between LDHC and oxidative stress in osteoclasts, both in vitro and in vivo. However, there is still a lack of LDH inhibitors available in the clinic, and further research into the role of LDH is needed.

We demonstrated the potential osteoprotective effects and protein targets of Tan IIA. Tan IIA can significantly ameliorate bone destruction by targeting LDHC to suppress oxidative stress and improve inflammation, ultimately suppressing osteoclast differentiation.

## Conclusion

**Fig. 7:**
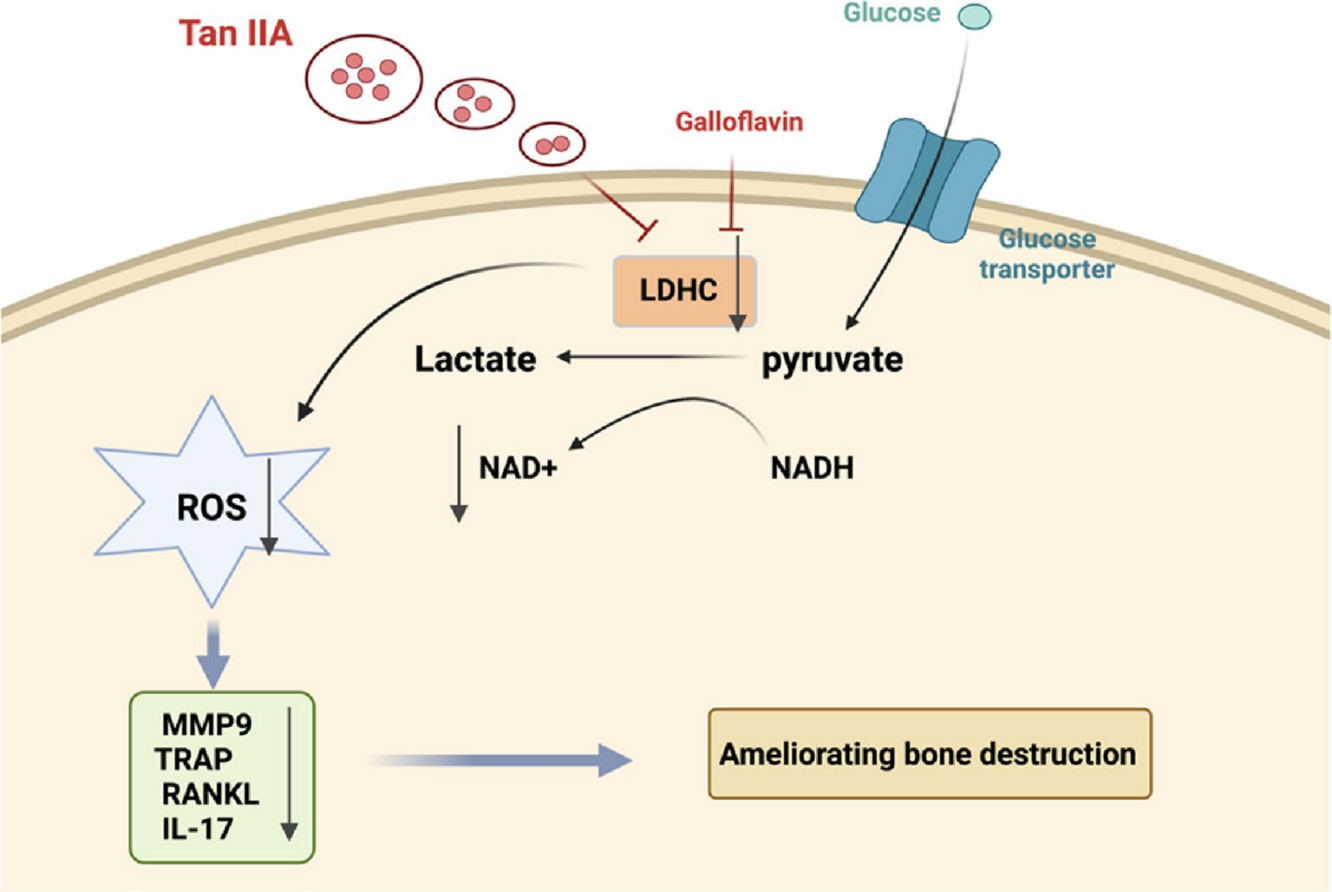
Schematic model showing the molecular mechanism of Tan IIA determined in this study. Tan IIA covalently binds to LDHC and inhibits its enzymatic activity, reducing the generation of NAD^+^, decreasing ROS accumulation and ultimately ameliorating bone destruction

Overall, our results showed that Tan IIA could covalently bind to LDHC and inhibit its enzymatic activity, decreasing ROS accumulation and ultimately regulating antioxidant and anti-inflammatory activities (Fig. 7). The mechanism of Tan IIA should be further investigated, and we expect it to be an alternative therapy for the treatment of bone destruction in RA.

## Supplementary Information

**Additional file 1:** **Figure S1.** Gene ontology pathway enrichment analyses of DEGs.

## Data Availability

The corresponding author has access to the final trial dataset and contractual agreements.

## Abbreviations

RA: Rheumatoid arthritis
Tan IIA: Tanshinone IIA
ABPP: Activity-based protein analysis
LDHC: Lactate dehydrogenase subunit
ROS: Reactive oxygen species
RANKL: Receptor activator of nuclear factor κB ligand
FLSs: Fibroblast-like synoviocytes
AIA: Adjuvant–induced arthritis
MTX: Methotrexate
CFA: Complete Freund’s adjuvant
EDTA: Ethylenediaminetetraacetic acid
HE: Haematoxylin–eosin staining
BV: Bone volume
TV: Total volume
Tb. N: Trabecular number
BS/BV: Bone surface ratio
Tb. Th: Trabecular thickness
MMP-9: Matrix Metalloproteinase 9
TRAP: Tartrate Acid Phosphatase
CTSK: Tissue Proteinase K
IL-17: Interleukin 17
FBS: Foetal bovine serum
BSA: Bovine serum albumin
DBIA: Desthiobiotin iodoacetamide
LDH: Lactate dehydrogenase
