# Supplementary material for: Tanshinone IIA inhibits osteoclastogenesis in rheumatoid arthritis via LDHC-regulated ROS generation
Source: Chin Med. 2023 May 15;18:54. doi: 10.1186/s13020-023-00765-1 (PMC10184368; doi:10.1186/s13020-023-00765-1)
Supplement: Supplementary file 1 — Additional file 1: Figure S1. Gene ontology pathway enrichment analyses of DEGs. [file 13020_2023_765_MOESM1_ESM.docx]

**Materials and methods**

- The method of AIA induction^[1]^

Besides the normal control group, the AIA model group, Tan IIA high, middle and low dosage groups and MTX group. Rats were injected with 0.1 mL complete Freund's adjuvant at the tail base to induce arthritis.

- Paw Swelling^[2]^

The ipsilateral hindfoot thickness of rats in each group was measured using vernier calipers, with repeated three times.

- Rats' pain domain^[3]^

Rats' pain domain testing was performed during the day portion of the circadian cycle only (06:00-18:00 h). Rats were placed in a plastic cage with a wire mesh bottom which al- lowed full access to the paws. Behavioral accommodation was allowed for approximately 15 min, until cage exploration and major grooming activities ceased. The area tested was the mid-plantar left hind paw, in the sciatic nerve distribution, avoiding the less sensitive tori (footpads). The paw was touched with 1 of a series of 8 von Frey hairs with logarithmically incremental stiffness (0.41, 0.70, 1.20, 2.00, 3.63, 5.50, 8.50, and 15.10 g) (Stoelting). The von Frey hair was presented perpendicular to the plantar surface with sufficient force to cause slight buckling against the paw, and held for approximately 6-8 s. Stimuli were presented at intervals of several seconds, allowing for apparent reso- lution of any behavioral responses to previous stimuli. A positive response was noted if the paw was sharply withdrawn. Flinching immediately upon removal of the hair was also considered a positive response. Ambula- tion was considered an ambiguous response, and in such cases the stimulus was repeated.

- The measurement of histopathological scores^[4]^

Histopathological characteristics were evaluated blindly, where grade 1 = hyperplasia of the synovial membrane and presence of polymorphonuclear infiltrates; grade 2 = pannus and fibrous tissue formation as well as focal subchondral bone erosion; grade 3 = articular cartilage destruction and bone erosion; and grade 4 = extensive articular cartilage destruction and bone erosio

**Figures**


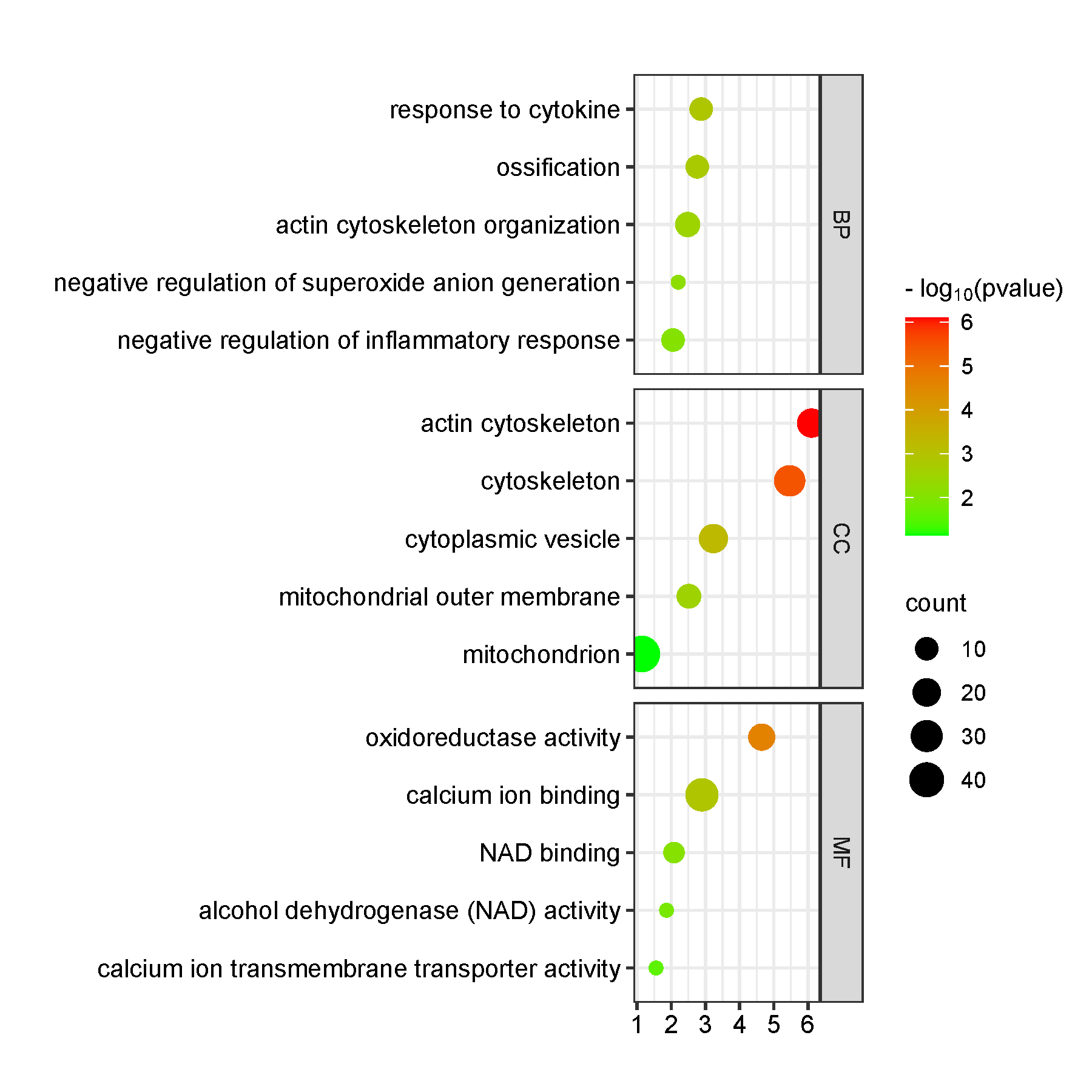


Figure S1. Gene ontology pathway enrichment analyses of DEGs. Top 5 terms of three category (BP, CC, MF) are drawn in a bubbler chart.

**References**

[1] LI W, WANG K, LIU Y, et al. A Novel Drug Combination of Mangiferin and Cinnamic Acid Alleviates Rheumatoid Arthritis by Inhibiting TLR4/NFκB/NLRP3 Activation-Induced Pyroptosis [J]. Front Immunol, 2022, 13(912933.

[2] CARDOSO R S, MESSORA M R, SILVA P H F, et al. Effects of Bifidobacterium animalis subsp. lactis HN019 on ligature-induced periodontitis in rats with experimental rheumatoid arthritis [J]. Beneficial microbes, 2020, 11(1): 33-46.

[3] CHAPLAN S R, BACH F W, POGREL J W, et al. Quantitative assessment of tactile allodynia in the rat paw [J]. Journal of neuroscience methods, 1994, 53(1): 55-63.

[4] GUO Q, ZHENG K, FAN D, et al. Wu-Tou Decoction in Rheumatoid Arthritis: Integrating Network Pharmacology and In Vivo Pharmacological Evaluation [J]. Front Pharmacol, 2017, 8(230.
